# Mini-Review: Immunogenic epitopes in apolipoprotein B-100 for atheroprotective immunization

**DOI:** 10.3389/fcvm.2024.1448664

**Published:** 2024-08-15

**Authors:** Norbert Gerdes, Roland Klingenberg

**Affiliations:** ^1^ Division of Cardiology, Pulmonology, and Vascular Medicine, Medical Faculty and University Hospital, Heinrich Heine University, Düsseldorf, Germany; ^2^ Cardiovascular Research Institute Düsseldorf (CARID), Medical Faculty, Heinrich Heine University, Düsseldorf, Germany; ^3^ Cardiology, Kerckhoff-Klinik, Bad Nauheim, Campus of the Justus-Liebig-University Giessen, Giessen, Germany; ^4^ Department of Cardiology, German Center for Cardiovascular Research (DZHK), Partner Site Rhine-Main, Bad Nauheim, Germany

**Keywords:** atherosclerosis, lipoproteins, immunity, immunization, inflammation

## Abstract

Here, we provide a concise overview of recent developments in the identification of immunogenic epitopes in human apolipoprotein B-100 for immunization against atherosclerotic cardiovascular disease. Major steps forward toward a clinical application of vaccines include the design of humanized mouse models, tetramer-based identification of antigen-specific T cells, and novel analysis tools, such as single-cell RNA sequencing and cytometry by time of flight, to assess temporal and spatial changes in immune cells in atherosclerotic cardiovascular disease.

## Graphical Abstract

Humanized mouse models enable comprehensive *in vivo* assessment of immune responses upon apolipoprotein B-100 (apoB-100) vaccination in the presence of constitutive apoB-100 expression, thus reflecting the human situation. Major histocompatibility class-tetramers identify immunogenic peptide epitopes in apoB-100 (orange) binding to the antigen-specific groove in HLA-DR (blue), with the latter coupled to biotin (yellow). Streptavidin (red) conjugation of this antigen-loaded complex with fluorochrome/lanthanide metals (yellow triangle) bound to the streptavidin core enables the detection of the tetramer. Single-cell RNA sequencing (scRNAseq) and the specific application of mass cytometry by time of flight along with vascular imaging by fluorodeoxyglucose positron emission tomography allow the vaccine response against atherosclerotic vascular disease in humans to be monitored.

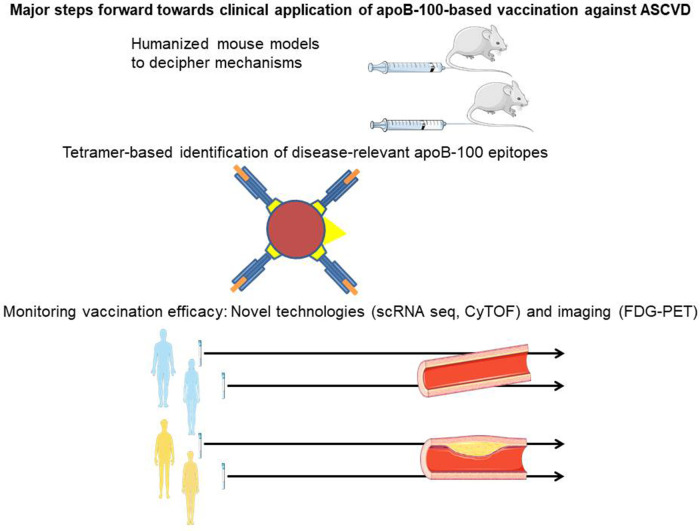

## Introduction

Apolipoprotein B-100 (apoB-100) constitutes the core protein in non-high-density lipoprotein (non-HDL) cholesterol (1, 2). The ApoB:ApoA-1 ratio was identified as a major predictor of adverse clinical events among several other classical cardiovascular risk factors in a primary prevention setting (3). In a meta-analysis of patients with atherosclerotic cardiovascular disease (ASCVD) treated with statins, apoB-100 remained a strong predictor of adverse clinical events (4).

The intricate link between innate and adaptive immunity in low-density lipoprotein (LDL)-driven atherosclerosis has long been recognized (5). Among findings bolstering this hypothesis is the discovery of a danger-associated peptide from apoB-100 (ApoBDS-1) that triggers innate proatherogenic responses (6) representing an immune reaction against self. Conversely, immune reactions directed against oxidized phospholipids or aldehydes covalently linked to the apoB molecule (modified peptides from apoB) have been identified as danger-associated molecular patterns (DAMPs) that are recognized by pattern recognition receptors (PRR) of innate immunity (7). This notion is supported by the finding that major histocompatibility class (MHC) class II-restricted antigen presentation by plasmacytoid dendritic cells, a cell type combining innate and adaptive immune responses, drives proatherogenic CD4+ T-cell immunity (8).

## Antibodies directed against immunogenic epitopes in human apolipoprotein B-100

Humoral immune responses comprise immunoglobulins IgG and IgM produced by B cells. Natural IgM antibodies are secreted by B1 cells, conferring atheroprotection (9). These antibodies recognize oxidation-specific epitopes in oxidized (ox)LDL, and immunization-mediated artificial expansion of IgM antibodies specific for one of these epitopes, oxidized phosphatidylcholine (PC), significantly reduced atherosclerosis in hyperlipidemic mice (10). Antibody library screening from patients with ASCVD events identified specific apoB-100 peptide sequences termed p210 and p45 (11). The prognostic accuracy of p210 was evaluated in the Malmö Diet and Cancer Cohort (*n* = 5,393, with a follow-up of more than 15 years) and demonstrated that higher IgG titers against native p210 conferred protection against coronary events [adjusted hazard ratio (95% confidence interval), 0.73 (0.56–0.97); *P* = 0.029] (12).

A novel humanized hypercholesterolemic mouse model of atherosclerosis characterized by T-cell receptor (TCR) transgenic T cells enabled the analysis of T-cell reactivity to human apoB-100 (hybridoma-derived) in human APOB100-tg *Ldlr*^tm1Her^ (HuBL) mice (13). Upon antigen injection, LDL induced a T-cell-dependent B-cell response, leading to the production of anti-LDL antibodies (IgG) that increased LDL clearance and ameliorated atherosclerosis (14). In another experiment using these TCR-transgenic humanized mice (BT1xHuBL cross), LDL-autoreactive T cells that provided T-cell help for anti-LDL antibody production conferred atheroprotection from birth (14).

## Human apolipoprotein B-100-specific atheroprotection mediated by regulatory T cells

Regulatory T cells (Tregs) constitute an inherent anti-inflammatory counterbalance to proatherogenic T-cell immunity (15). The transcription factor FoxP3 constitutes a key lineage marker and master switch in the regulation and development of Tregs that centrally mediates peripheral tolerance (antigen-specific protection from autoimmunity) (16–18). Natural thymus-derived Tregs characterized by the surface markers CD4 and CD25 (19, 20), or more specifically by the expression of CD4 with the *bona fide* marker of Tregs, Foxp3, confer atheroprotection in hyperlipidemic mice (21). The depletion of transgenic Tregs exacerbates atherosclerosis in hyperlipidemic mice and promotes hypercholesterolemia [very-low-density lipoprotein (VLDL)/chylomicron fraction] (21). In patients with acute coronary syndrome, Tregs (CD4+CD25+CD127low) were enriched in coronary thrombi compared with peripheral blood mononuclear cells. A DNA sequencing approach identified the clonal restriction of T cells, indicating a reduced T-cell receptor diversity in coronary thrombi (22).

**Figure 1:**
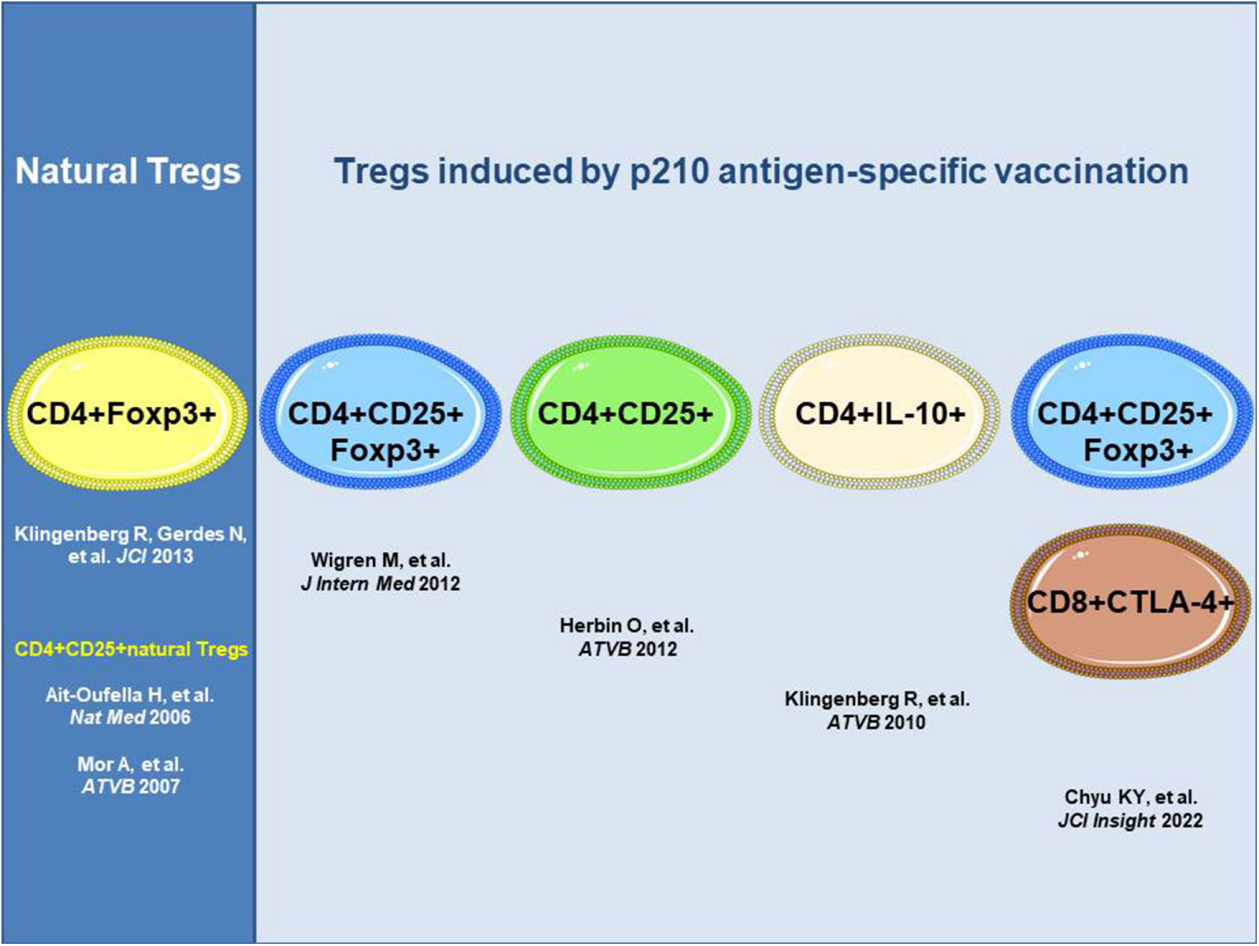
ApoB-100 p210 peptide vaccination induces atheroprotective regulatory T cells. All approaches shown used apoB-100 peptide p210 as a vaccine antigen; however, different routes and adjuvants impact distinct mechanisms of action with distinct subsets of the Tregs involved. ApoB-100, apolipoprotein B-100; CD4+ T cell, cluster of differentiation 4 positive T cell; CTLA-4, cytotoxic T-lymphocyte-associated protein-4; Foxp3, forkhead box P3; IL-10, interleukin-10.

The antigen-specific induction of atheroprotective immunity against immunogenic epitopes in human apoB-100 mediated by inducible Tregs has been shown for the apoB-100 peptide p210 identified by human antibody library screening in patients with ASCVD (11). Distinct subsets of inducible Tregs have been detected with different routes of administration of the p210 vaccine and adjuvants that effected distinct mechanisms of action, all of which resulted in a reduction in atherosclerosis in hyperlipidemic mice (Figure 1). These distinct subsets comprise CD4+Foxp3+cells producing transforming growth factor beta (TGFβ) upon subcutaneous administration of the antigen (23), CD4+Foxp3+ cells upon subcutaneous infusion of adjuvant-free antigen (24), or CD4+IL-10+ regulatory type 1 (Tr1) cells producing IL-10 upon nasal administration of the p210 antigen coupled to the cholera toxin B subunit as an adjuvant with apoB-100-specific attenuation of cell proliferation in an antigen rechallenge assay (25). Recently, subcutaneous immunization with the p210 antigen in peptide amphiphile micelles (nanoparticles) was shown to mediate atheroprotection with an increase in CD8+CTLA4+ Tregs recognizing the epitope bound to MHC class I in mice along with CD4+CD25+Foxp3+ Tregs (26).

The repetitive adoptive transfer of naive CD4+ T cells recognizing human ApoB100 from TCR-transgenic mice into humanized hypercholesterolemic mice with TCR-transgenic T cells against human apoB-100 (13) induced tolerance in recipient mice with an increase in Tr1 cells that suppress apoB-100-specific responses via IL-10 (27).

Tetramers constructed to contain a streptavidin-biotin core labeled with a fluorochrome coupled to four HLA-DR molecules carrying a defined antigen (here the apoB peptide p18) allow the detection of apoB peptide-specific CD4+ T cells in human peripheral blood mononuclear cells (28). Atherosclerosis in apolipoprotein E-deficient (*Apoe*^−/−^) mice vaccinated with p18 (identical sequence in human and mouse) reduced atherosclerosis. Interestingly, Tregs co-expressing the lineage-specific transcription factors ROR*γ*t or T-bet were detected, suggesting that phenotype changes occur when cardiovascular disease is present or evolving (28). In mice, MHC class II tetramers were employed to detect CD4+ T cells that recognize the peptide apo B978-993 (p6) (29). Using DNA sequencing, a population of oligoclonal apoB-100-reactive CD4+ T cells (apoB+) was found to reside in the lymph nodes of healthy C57Bl/6 mice (29). Of note, apoB-100-reactive CD4+ Tregs change in the course of developing murine atherosclerosis, acquiring a pro-inflammatory T helper 17/T helper 1 phenotype. In patients with coronary artery disease, apoB-100-reactive T cells were found and are characterized by distinct features, including the secretion of Th1 and Th17 cytokines (29), which is in line with the notion of temporal changes in the Treg phenotype and function.

A major step forward was the unbiased identification of immunogenic apoB-100 sequences using apoB-100 tetramer constructs, which yielded six immunodominant HLA-II-restricted apoB-100 epitopes in humans that trigger highly specific immune responses correlated with coronary artery disease severity (30). The activation of CD4+ T cells was associated with the severity of coronary artery disease, characterized by the skewed expression of antigen-experienced phenotypes and an increased secretion of pro-inflammatory cytokines in response to these dominant apoB-100 epitopes (30).

Technological advances comprising multiplex immune phenotyping to decipher plaque immune cell heterogeneity, single-cell RNA sequencing (scRNAseq), and the specific application of mass cytometry by time of flight (CyTOF) have enabled the comprehensive characterization of single (immune) cells residing in the atherosclerotic plaque and peripheral blood. By employing these technologies, a recent study found that a high frequency of memory T cells is associated with protection against myocardial infarction or stroke after carotid endarterectomy (31). A recent report demonstrated that full-length transcriptome analysis of individual pairs of TCR *α* (V and J segment) and *β* chain (V and J segment) sequences revealed that CD4+ T cells carrying this combination were specifically expanded upon immunization with the specific apoB-100 peptide p6. Furthermore, clonally expanded T cells expressed a clear Treg signature by scRNAseq with upregulation of suppressor genes after immunization with the p6 apoB-100 peptide (32).

## Outlook

A number of recent technological advances have enabled major steps to be taken toward the clinical application of apoB-100-based immunization against ASCVD in humans. One of these advances is the development of humanized mouse models expressing human apoB-100, which allow a comprehensive analysis of the distinct parts of adaptive (humoral and cellular) immunity over time, mirroring the situation in humans. Tetramer-based recognition of antigen-specific T cells has identified a set of peptide sequences within apoB-100 that are immunogenic in humans and associated with the severity of coronary artery disease. To assess their potential use in vaccination against ASCVD in humans, further studies analyzing individuals at different stages and various vascular beds of ASCVD are needed. It will be important to determine the optimal vaccination setting and strategy by addressing aspects such as combining several apoB-100 antigens vs. single peptide vaccination, the type of adjuvant, and the route and timing of vaccination. scRNAseq and the specific application of mass CyTOF have made it possible to assess temporal and spatial changes in immune cells in ASCVD, adding a new level of complexity but also the ability to distinguish specific aspects of apoB-100 vaccination. Finally, these novel technologies will assist in deciphering the mechanisms involved and the specific roles of subsets of Tregs and B cells or antibodies and should also help to assess the efficacy of novel therapeutic approaches. Future studies need to establish the optimal read-out after apoB-100 vaccination to allow for quantitative analysis over time, making use of tetramers, scRNAseq, CyTOF, and vascular imaging strategies such as fluorodeoxyglucose positron emission tomography to determine efficacy and potential side effects.
